# Stability of histone post-translational modifications in samples derived from liver tissue and primary hepatic cells

**DOI:** 10.1371/journal.pone.0203351

**Published:** 2018-09-07

**Authors:** Philip A. Gruppuso, Joan M. Boylan, Valerie Zabala, Nicola Neretti, Nebiyu A. Abshiru, Jacek W. Sikora, Emma H. Doud, Jeannie M. Camarillo, Paul M. Thomas, Neil L. Kelleher, Jennifer A. Sanders

**Affiliations:** 1 Department of Pediatrics, Brown University and Rhode Island Hospital, Providence, RI, United States of America; 2 Department of Molecular Biology, Cell Biology, and Biochemistry, Brown University, Providence, RI, United States of America; 3 National Resource for Translational and Developmental Proteomics, Northwestern University, Evanston, Illinois, United States of America; 4 Department of Molecular Biosciences, Northwestern University, Evanston, Illinois, United States of America; 5 Division of Hematology/Oncology, Feinberg School of Medicine, Northwestern University, Chicago, Illinois, United States of America; 6 Department of Chemistry, Northwestern University, Evanston, Illinois, United States of America; 7 Department of Pathology and Laboratory Medicine, Brown University, Providence, RI, United States of America; Ludwig-Maximilians-Universitat Munchen Adolf-Butenandt-Institut, GERMANY

## Abstract

Chromatin structure, a key contributor to the regulation of gene expression, is modulated by a broad array of histone post-translational modifications (PTMs). Taken together, these “histone marks” comprise what is often referred to as the “histone code”. The quantitative analysis of histone PTMs by mass spectrometry (MS) offers the ability to examine the response of the histone code to physiological signals. However, few studies have examined the stability of histone PTMs through the process of isolating and culturing primary cells. To address this, we used bottom-up, MS-based analysis of histone PTMs in liver, freshly isolated hepatocytes, and cultured hepatocytes from adult male Fisher F344 rats. Correlations between liver, freshly isolated cells, and primary cultures were generally high, with R^2^ values exceeding 0.9. However, a number of acetylation marks, including those on H2A K9, H2A1 K13, H3 K4, H3 K14, H4 K8, H4 K12 and H4 K16 differed significantly among the three sources. Inducing proliferation of primary adult hepatocytes in culture affected several marks on histones H3.1/3.2 and H4. We conclude that hepatocyte isolation, culturing and cell cycle status all contribute to steady-state changes in the levels of a number of histone PTMs, indicating changes in histone marks that are rapidly induced in response to alterations in the cellular milieu. This has implications for studies aimed at assigning biological significance to histone modifications in tumors versus cancer cells, the developmental behavior of stem cells, and the attribution of changes in histone PTMs to altered cell metabolism.

## Introduction

Nucleosomes, the basic repeating units of eukaryotic chromatin, are formed by the wrapping of DNA around histone octamers composed of two copies of each of the four core histones: H2A, H2B, H3 and H4 [1]. Interaction of DNA between nucleosomes and histone H1 mediates additional chromatin folding [1]. The structure of chromatin plays an important role in the regulation of gene expression by determining the accessibility of specific regions of DNA [2]. Critical to this relationship between chromatin structure and gene expression is a broad array of post-translational modifications (PTMs) to which histones are subject [3]. Among the types of histone modifications are phosphorylation, acetylation, methylation and monoubiquitylation. Methylation occurs as mono-, di- or trimethylation. Taken together, the modifications at specific histone sites are often referred to as the “histone code”. As per a recent survey of the literature, known histone modifications occur at over two hundred and thirty sites, and total more than five hundred [4], offering the potential for extraordinary combinatorial modification and highly complex regulation of gene expression.

Since the identification of the relationship between histone modifications, chromatin structure and epigenetics, antibody-based analysis of histone modifications has been the standard. However, antibody specificity is an issue owing to the similarities among modifications (mono-, di- and trimethylation), similarity of primary structure among various sites on what are often related histone isoforms, and interactions between sites that affect antibody binding. The quantitative analysis of histone PTMs by mass spectrometry (MS) has more recently provided for the surveying of numerous histone marks and their combinations [1]. Examples abound of MS-based strategies providing insight into the epigenotypes associated with the phenotypic characteristics of stem cells [5], epigenetic regulation during development [6], cell senescence [7], and, perhaps most notably, cancer pathophysiology [8].

Only a small number of studies have presented evidence for genome-wide changes in DNA methylation between primary cells and the tissue from which they were derived [9,10]. To our knowledge, only a single, recent study [11] focusing on several types of cancer and related primary cells and cell lines has systematically examined the effect of cell isolation and culture on histone posttranslational modifications. As we undertook studies on the role of histone modifications in determining the hepatocyte phenotype in the late gestation fetal rat [12,13], interpretation of our results required an understanding of the stability of histone modifications in hepatic cells under a variety of *in vivo* and *in vitro* conditions. The present report is an analysis of histone modifications in liver, freshly isolated hepatocytes and cultured hepatocytes, with an additional focus on changes related to altered cell cycle status.

## Materials and methods

### Materials

Collagenase/elastase and deoxyribonuclease were purchased from Worthington Biochemical Corporation (Lakewood NJ). Cell culture reagents, including Minimal Essential Media alpha, fetal bovine serum, antibiotic-antimycotic solution and amino acids, were purchased from Life Technologies (Carlsbad CA). Hank’s buffered salt solution and other chemicals were acquired from Sigma (St Louis MO). Epidermal growth factor (EGF) and porcine insulin were purchased from PeproTech (Rocky Hill NJ).

### Animals

All animals were euthanized by exsanguination under general anesthesia. Animal studies were performed in accordance with the guidelines of the National Institutes of Health and were approved by the Rhode Island Hospital Institutional Animal Care and Use Committee. Adult male Fisher F344 rats were obtained from Charles River Laboratories, Wilmington, MA. All animals were housed under standard conditions with access to food and water ad libitum. Three biological replicates per condition were used for all studies. Adult liver tissue was obtained under isoflurane anesthesia. Hepatocytes were isolated from adult rats using a 2-step collagenase digestion procedure [14].

### Hepatocyte isolation and culture

Adult hepatocytes were isolated and cultured under defined, serum-free conditions as per methods standard to our laboratory [15,16]. Where indicated, cultured adult hepatocytes were induced to proliferate by adding epidermal growth factor (EGF) and insulin (both at 100 ng/mL) to the culture media [16] for 0, 2, 6, 24, 48 or 72 hr. Dosing was performed so that hepatocytes in different experimental groups were maintained in culture for 72 hr. That is, EGF and insulin were added 2, 6, 24, 48 or 72 hr before all cells were lysed at the end of the experiment. For all cell experiments, cells were flash frozen in liquid nitrogen at the end of the experiments and stored at -80°C.

### Preparation of liver and hepatocyte histones

Nuclei were isolated from adult rat liver as previously described [17]. The nuclear pellet was washed twice in Buffer B (15mM HEPES, pH 7.5, 30% sucrose, 60mM KCl, 15mM NaCl, 2mM EDTA, 0.5mM EGTA, 14mM 2-mercaptoethanol with freshly added 10mM sodium butyrate, 10μg/mL leupeptin, 10μg/mL aprotinin and 24.4μg/mL 4-(2-aminoethylbenzenesulfonyl fluoride) hydrochloride. Nuclei were isolated from frozen cell pellets [18]. Histones were extracted from isolated nuclei using 0.4N sulfuric acid and were chemically derivatized using propionic anhydride and digested with trypsin [19].

All studies were carried out on triplicate biological samples and acquired as described below in technical triplicate (or duplicate). When technical replicates were performed, results were averaged to yield a single mean for each biological replicate.

### Histone PTM LC-MS analysis

Histone peptides were resuspended in 0.1% trifluoroacetic acid prior to mass spectrometric analysis on a ThermoFisher Scientific TSQ Quantum QqQ MS (San Jose CA). Using a Dionex RSLCnano system (Sunnyvale CA), peptides were loaded onto a trapping column (3cm x 150μm) and separated on a PicoChip analytical column (10cm x 75μm), both packed with ProntoSIL C18-AQ, 3μm, 200Å pore size (New Objective, Woburn MA). Elution occurred over a chromatography gradient of buffer B from 0 to 35% at a flow rate of 0.30 μL/min over 45 minutes. Solvent A: 0.1% formic acid in water, and B: 0.1% formic acid in 95% acetonitrile. The peptides were then introduced into the QqQ MS by electrospray from an integrated emitter with 10 μm tip (New Objective) after elution from the analytical column. Targeted analysis of unmodified and various modified histone peptides was performed with the following settings: collision gas pressure of 1.5 mTorr; Q1 peak width of 0.7 (FWHM); cycle time of 3 s; skimmer offset of 10 V; electrospray voltage of 2.5 kV. Histone peptides (modified and unmodified) were selected for monitoring based on previous publications [20,21]. A list of all peptides monitored in the assay can be found in S1 Table.

### MS data analysis

Raw MS files were imported and analyzed in Skyline with Savitzky-Golay smoothing [22]. All Skyline peak area assignments were manually confirmed. The peak areas determined by Skyline (S2 Table) were imported into an in-house built software platform to determine the percent relative abundance for every modification state for each residue of interest. The percent relative abundance was calculated by dividing the peak area for a given modification state (i.e. H3 K4Me3) by the sum of the peak areas for all peptides containing that residue (i.e., H3 K4Un, H3 K4Me1, H3 K4Me2, H3 K4Me3, and H3 K4Ac). For peptides containing multiple sites of modification (i.e., H3 K9/K14), the relative abundance was determined by dividing the sum of the peak areas for all peptides with a given modification. For instance, to determine the relative amount of H3 K9Ac, the summed intensities of those peptide forms containing H3 K9Ac (i.e., H3 K9AcK14Un and H3 K9AcK14Ac) are divided by the by the sum of all ten peptides containing those two residues (i.e., H3 K9UnK14Un, H3 K9Me1K14Un, H3 K9Me2K14Un, H3 K9Me3K14Un, H3 K9AcK14Un, H3 K9UnK14Ac, H3 K9me1K14Ac, H3 K9Me2K14Ac, H3 K9Me3K14Ac, and H3 K9AcK14Ac). The relative abundance data for all experiments are provided in S3 Table.

### Statistical analysis

**Fig 1:**
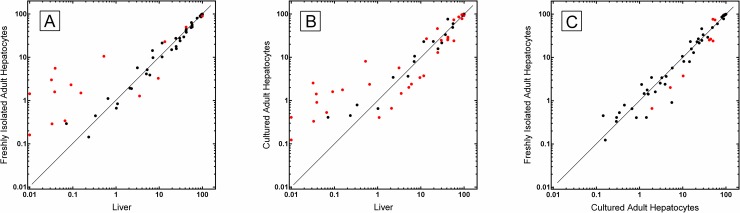
Correlation of histone post-translational modifications from various biological sources. The graphs depict the relative abundance of histone modifications in nuclei derived from adult rat liver tissue, freshly isolated adult rat hepatocytes, and adult rat hepatocytes maintained in culture for 72hr prior to harvesting. Each point represents the mean of one histone mark measured in three biological replicates per biological source. The solid lines are the lines of identity for each graph. Red symbols are data points for which the statistical analysis showed a significant difference between the two biological sources shown in that graph. Panel A. Liver versus freshly isolated hepatocytes. Panel B. Liver versus cultured hepatocytes. Panel C. Cultured hepatocytes versus freshly isolated hepatocytes.

The correlation statistics (correlation coefficients and 95% confidence intervals) for the liver and hepatocyte data were generated using the linear regression analysis function in GraphPad Prism 6.05 (GraphPad Software, La Jolla, CA). One-way ANOVA was performed on pairwise comparisons of groups and across individual marks in all three groups. Changes in abundance of histone marks and their statistical significance (false discovery rate [FDR] adjusted p-values) were determined using the empirical Bayes method within the R package limma [23]. The liver-fresh hepatocyte-cultured hepatocyte comparisons were carried out using two complementary methods. For the correlation analyses in Fig 1, we performed pair-wise post-hoc analyses. The analyses focusing on specific histone modifications utilized a post-hoc analysis across all three biological sample groups.

## Results

Histones undergo modifications in response to a number of environmental factors, among which are metabolites that serve as either cosubstrates for histone modifying enzymes, metabolites that are modulators of these enzymes [24], and cell signaling pathways responsive to extracellular signaling molecules [25]. We therefore hypothesized that changes in the cellular environment that occur during hepatocyte isolation and culture could result in changes in histone PTMs. To test this hypothesis, we analyzed histone preparations derived from adult rat hepatocytes that were freshly isolated or cultured under defined conditions for 72 hr. Histones prepared from liver tissue were also studied as a condition reflecting the *in vivo* metabolic and hormonal milieu and the microenvironment in which hepatocytes reside in the intact liver. Results using the means of triplicate biological samples for each histone mark (Fig 1, S4 Table) showed that the relative abundance of histone PTMs was significantly correlated regardless of the biological source. The correlation varied to a modest degree when comparing liver to freshly isolated cells (R^2^ = 0.987) and freshly isolated cells to cultured cells (R^2^ = 0.965). The degree of correlation was marginally less for the comparison of cultured cells with liver (R^2^ = 0.944). However, ANOVA followed by post-hoc pair-wise analyses (Fig 1, S5 Table) showed a clear effect of biological source. Of the 59 histone marks quantified in the analysis of all three biological sources, the comparison of liver with freshly isolated cells yielded 15 that differed significantly between the two. These altered marks represented a mix of unmodified, methylated and acetylated sites. The comparison of freshly isolated versus cultured hepatocytes showed only nine marks that differed significantly. In this case, the differences reflecting altered acetylation. In contrast, the comparison of liver with cultured hepatocytes identified significant differences for 35 of the 59 marks that were quantified, reflecting a mix of unmodified, methylated and acetylated sites.

**Fig 2:**
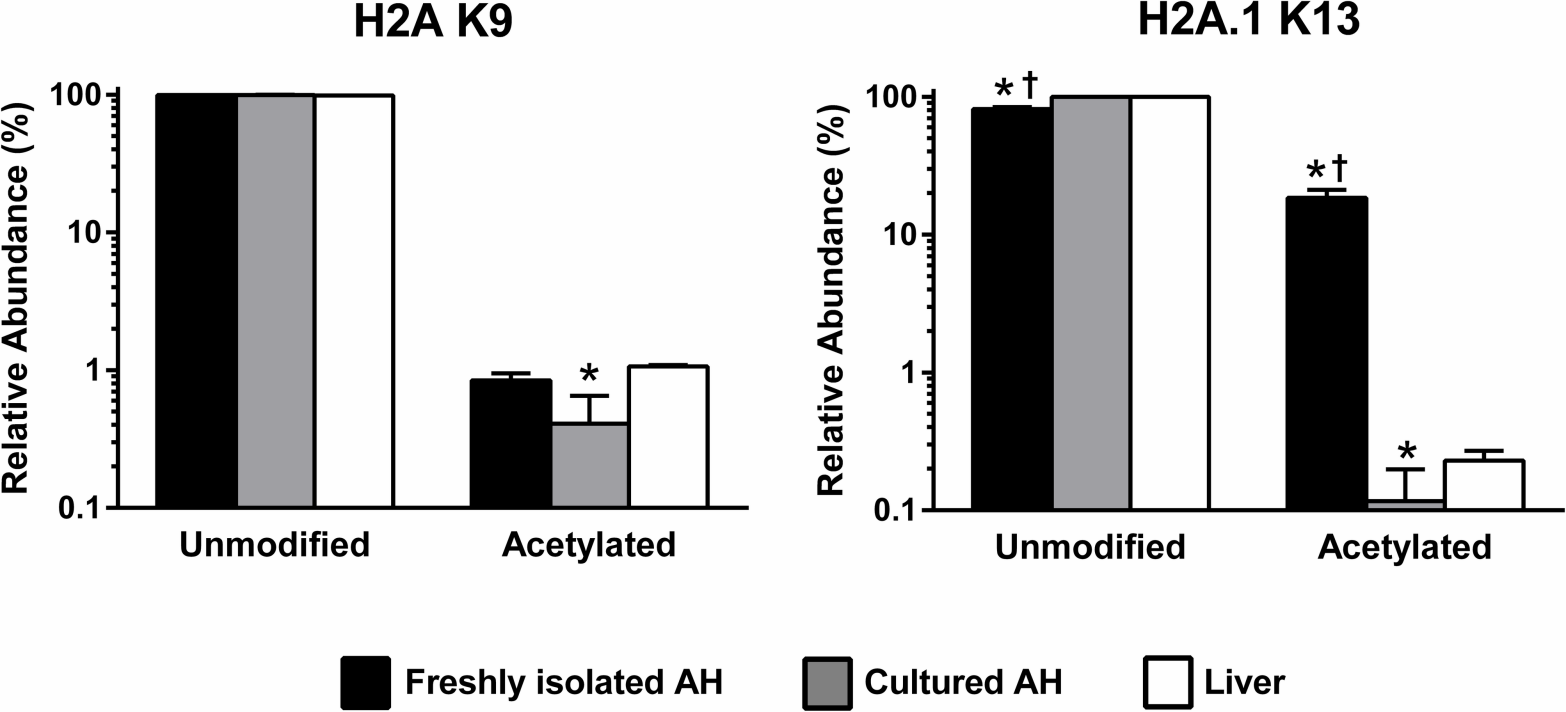
Relative abundance of modifications of sites on histones H2A and H2A1. Data, shown as the mean and standard deviation, represent the relative abundance of unmodified and acetylated residues in samples derived from freshly isolated adult hepatocytes (AH; black fill), cultured AH (grey fill) or liver tissue (white fill). *, significant versus liver; †, significant versus cultured adult hepatocytes.

Based on these results, we more closely examined modifications at specific sites of histones derived from the three biological sources, freshly isolated hepatocytes, cultured hepatocytes or liver. The K9 site on histone H2A showed a modest reduction in acetylation when hepatocytes were cultured for 72 hr (Fig 2). In contrast, the K13 site on histone H2A1 showed much higher acetylation in freshly isolated cells relative to liver or cultured hepatocytes (Fig 2).

**Fig 3:**
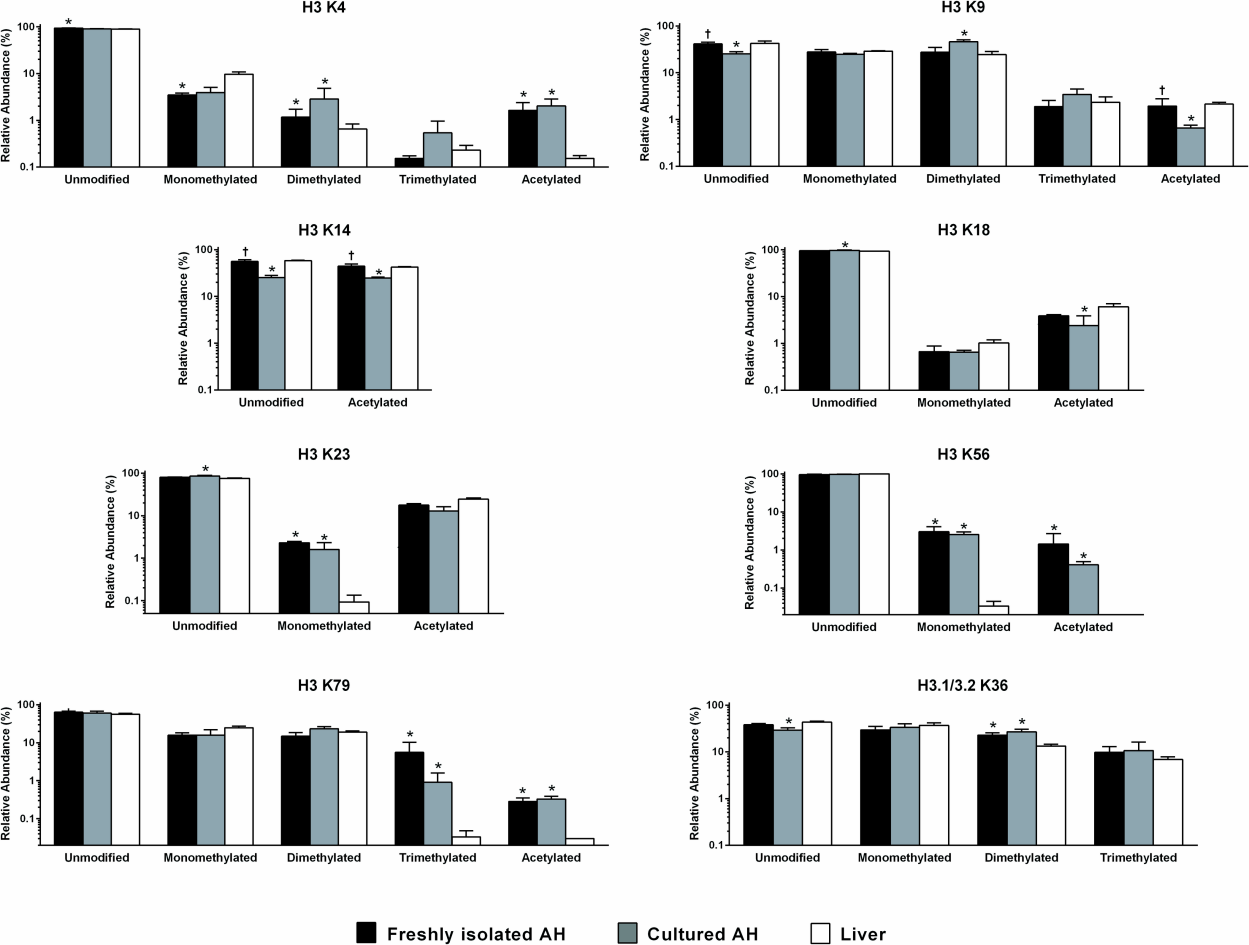
Relative abundance of modifications of sites on histones H3 and H3.1/3.2. Data, shown as the mean and standard deviation, represent the relative abundance of unmodified, monomethylated, dimethylated, trimethylated and acetylated residues in samples derived from freshly isolated adult hepatocytes (AH; black fill), cultured AH (grey fill) or liver tissue (white fill). *, significant versus liver; †, significant versus cultured adult hepatocytes.

A number of sites on histones H3 and H3.1/3.2 showed marked differences between isolated hepatocytes, cultured hepatocytes and liver (Fig 3). In some cases, such as H3 K4, the freshly isolated and cultured cells were concordant in showing significant differences from liver in both methylation and acetylation. In contrast, H3 K14 acetylation was nearly identical in liver and freshly isolated cells while cultured hepatocytes differed markedly from both.

**Fig 4:**
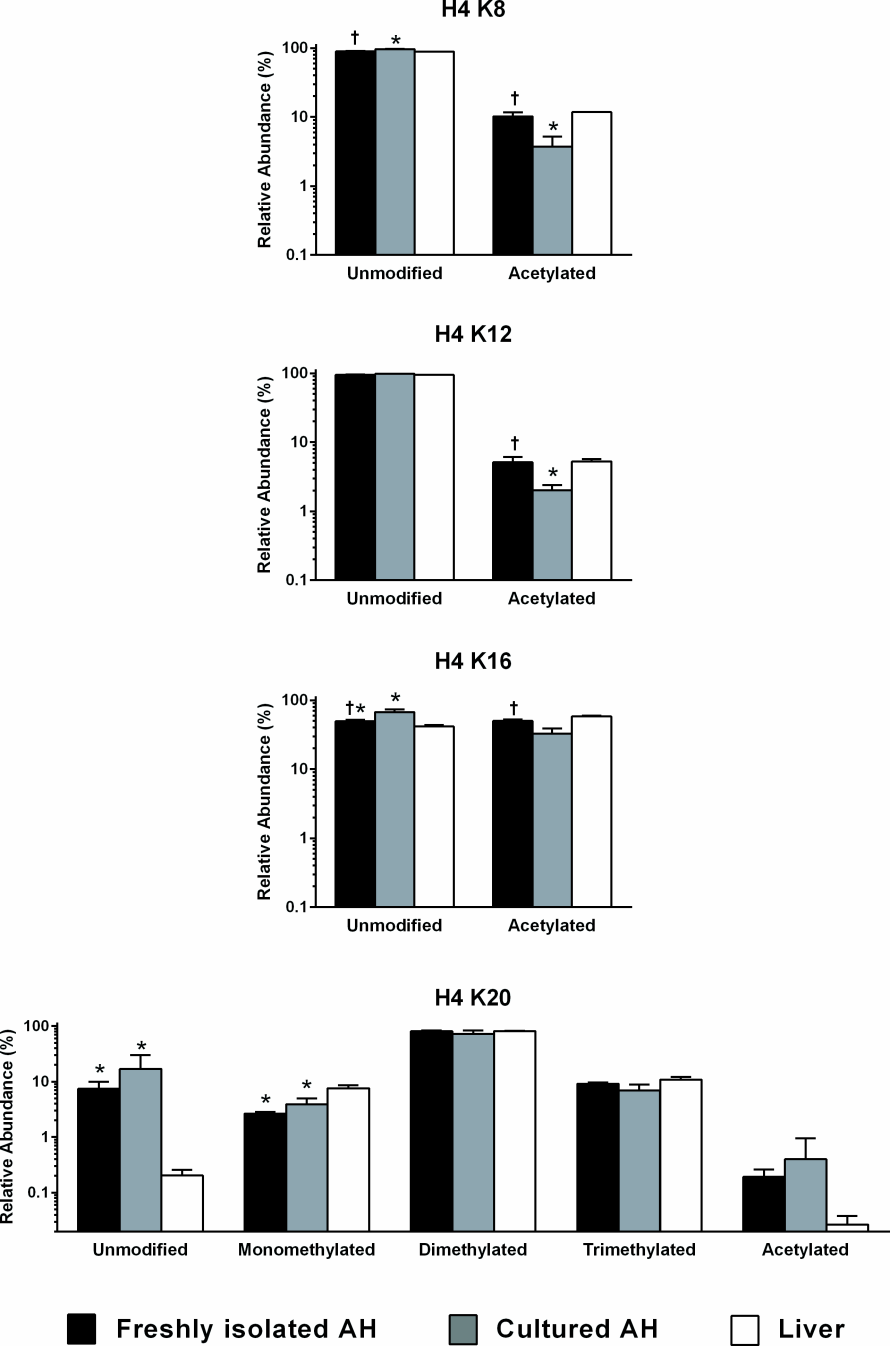
Relative abundance of modifications of sites on histone H4. Data, shown as the mean and standard deviation, represent the relative abundance of unmodified, monomethylated, dimethylated, trimethylated and acetylated residues in samples derived from freshly isolated adult hepatocytes (AH; black fill), cultured AH (grey fill) or liver tissue (white fill). *, significant versus liver; †, significant versus cultured adult hepatocytes.

Four sites on histone H4 showed variability among the three biological sources (Fig 4). Acetylation of the K8, K12 and K16 sites all showed significant differences between freshly isolated and cultured hepatocytes with the former more closely coinciding with the modifications on liver-derived histones. In contrast, H4 K20 showed a marked difference in the relative abundance of the unmodified site in liver samples relative to isolated or cultured hepatocytes.

To identify changes in adult rat hepatocyte histone PTMs associated with progression through the cell cycle, we cultured adult hepatocytes in the absence or presence of insulin plus EGF. These factors are able to induce the vast majority of cultured adult hepatocytes to enter and traverse the cell cycle *in vitro* [26]. For this experiment, we controlled for time in culture by harvesting cells in all experimental groups after 72 hr in culture. To generate data on various stages of the cell cycle, primary hepatocyte cultures were supplemented with EGF plus insulin 2, 6, 24, 48 or 72 hr prior to cell lysis. The 2 and 6 hr samples correspond to early G1. At 24 hr, the cells are traversing G1-S. Cells harvested at 48 hr have completed S-phase. By 72 hr, they have completed M and are beginning to again synthesize DNA [27,28].

**Fig 5:**
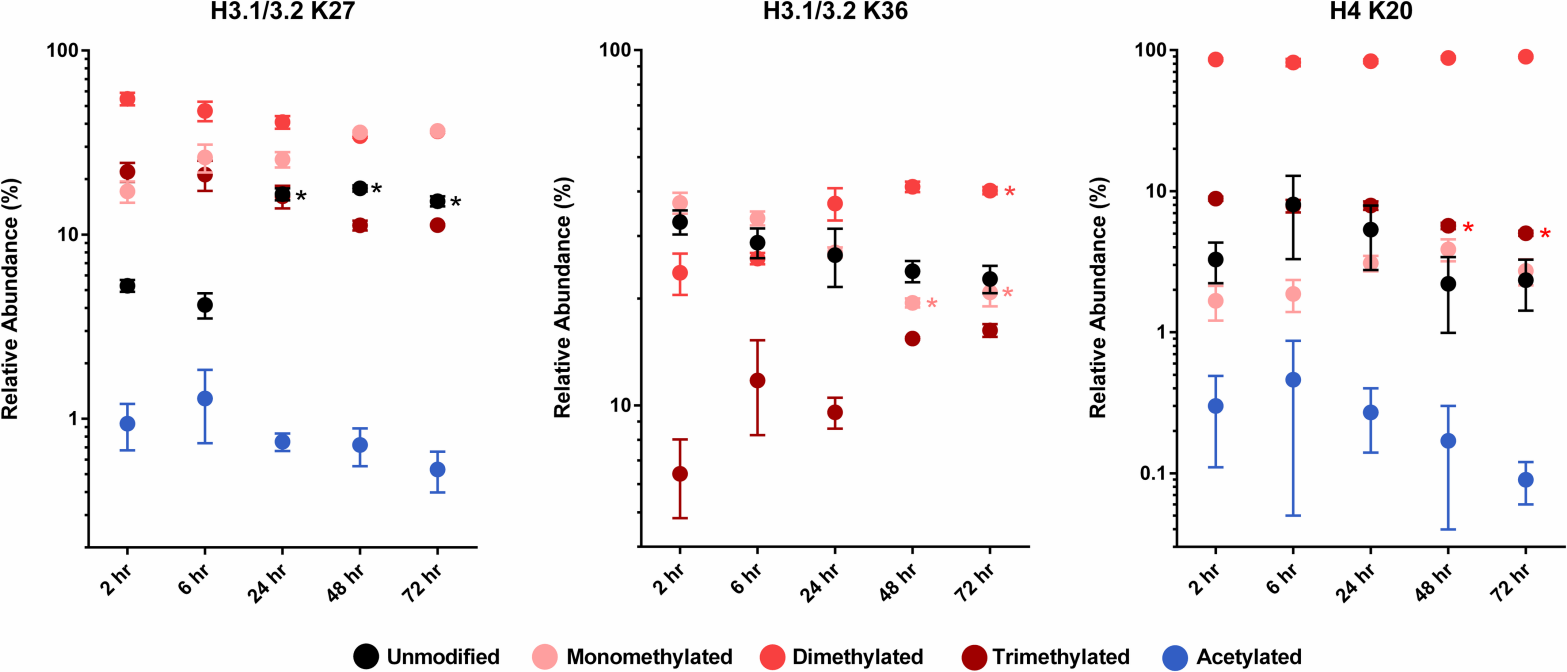
Relative abundance of selected histone modifications in synchronously proliferating, cultured adult hepatocytes. Data, shown as the mean and standard deviation, represent the relative abundance of unmodified, monomethylated, dimethylated, trimethylated and acetylated residues in samples derived from cultured adult hepatocytes. As described in *Methods*, the adult hepatocytes were stimulated to proliferate *in vitro*, then harvested to generate samples representing specific phases of the cell cycle. *, significant versus unstimulated adult hepatocytes maintained in culture for 72 hr.

Results (Fig 5) showed several changes. We observed significant alterations in two sites located on H3.1/3.2, K27 and K36, and on the K20 site in histone H4. Stringent statistical analysis of H3.1/3.2 K27 showed significant changes only in the unmodified site. However, the increase in the relative abundance of the unmodified site could be accounted for by declines in dimethylation, trimethylation and acetylation. In the case of the H3.1/3.2 K36 site, there was again a decline in the unmodified site, but it did not reach statistical significance. The observed changes in mono- and dimethylation were significant. The H4 K20 site showed a modest but significant decline in trimethylation that appeared to coincide with an increase in monomethylation.

## Discussion

The development of mass spectrometry-based technologies to quantify histone PTMs has opened the door to examining the role of this important epigenetic mechanism in human physiology and pathophysiology [1,29,30]. These methodologies have shown that the post-translational modification of histones is complex, with crosstalk between various sites and modifications [31], and highly dynamic [32]. Histone acetylation, which is more challenging given the multiple substrates that contribute to this PTM, has been recently found to be highly variable with regard to turnover rate [20]. The “bottom-up” approach used in the present studies, which involves proteolytic generation of peptides, allows a view of some combinatorial interactions between modification sites. A “top-down” approach, which allows for examination of intact proteins, allows for a more complete view of interactions between sites. However, the methodological and analytical challenges of the top-down approach have thus far prevented its general use for high-throughput histone analyses [29].

The critical role of histone modifications in the regulation of gene expression, as well as their dynamic nature, led us to explore their stability and propensity for change in several models of liver biology in the rat. Our laboratory’s interest in late gestation liver development and its relationship to hepatic carcinogenesis has focused, in part, on the regulation of gene expression [33,34,35,36,37]. Our studies have indicated that the mechanisms for nutrient-mediated transcriptional control in the developing liver are, to a large degree, independent of well characterized, canonical transcript factor signaling pathways. This observation has led to our interest in chromatin structure as a critical regulator of fetal hepatocyte gene expression and the related contribution of progenitor cells to hepatocellular carcinogenesis [33]. This, in turn, motivated our interest in histone modifications. The ability of the Kelleher laboratory to quantify the relative abundance of histone marks provided an opportunity to carry out studies on the physiologic and pathophysiologic contribution of histone PTMs to hepatocyte biology. However, we realized that we had to first characterize the histone PTM profile across standard models of hepatocyte biology in the rat.

The present studies demonstrate that acute hepatocyte isolation, the culturing of these cells, and their cell cycle status all contribute to steady-state changes in the levels of a number of histone PTMs. Comparing isolated and cultured rat hepatocyte samples with liver-derived histones, we observed substantial and statistically significant differences in a number of histone marks. Only a few sites and modifications, H2A K13, H4 K8, H4 K12 and H4 K16 acetylation, showed differences between acutely isolated and cultured cells. In studies on HeLa S3 cells [20], these sites varied considerably in their turnover rate and t_1/2_. However, the HeLa S3 findings do not seem to explain the changes we observed with acute cell isolation.

In contrast to site-specific acetylation, methylation marks were relatively stable when isolated hepatocytes were cultured for 72 hr. However, several sites on histones H3 and H4 showed differences in modifications when comparing hepatocytes with liver-derived histones. Among these were monomethylation of H3K4, H3K23, and H3K56, dimethylation of H3.1/3.2K36 and trimethylation of H3K79. A recent paper by Noberini et al. [11] describes a study similar to our own that focused on changes in the histone code associated with cell isolation and culture. Studying three representative tumor models, breast cancer, glioblastoma and ovarian cancer, these investigators found extensive changes in histone marks that were generally time dependent and apparent only in long-term culture of tumor cells. Among the changes they observed were decreases in H3 K27 di- and trimethylation, H3 K79 mono- and dimethylation, and H3 K9 and K 14 acetylation. Also observed was an increase in H3 K36 mono- and dimethylation. Several of these changes are consistent with our own data, a noteworthy observation given the difference in model systems and the performance of the analyses by two unrelated laboratories.

A caveat in the interpretation of these results is the cellular heterogeneity of liver tissue. Hepatocytes account for about 90% of the cell mass in adult liver [38]. However, the polyploid nature of these cells increases the relative contribution of hepatocyte histones to the total liver histone complement. Given these factors, we considered liver histone analyses to be largely reflective of hepatocyte histones.

As epigenetic marks, histone modifications must be maintained through the process of DNA replication and cell division. Consistent with this, most histone marks are unaltered as cells replicate, although the maintenance of histone marks during cell cycle progression is a complex conundrum. As discussed recently by Ma et al. [39], both histone marks and writer/reader complexes must be maintained as chromatin is replicated. Marks whose dynamics during cell cycle progression have been characterized include H3 K27Me3 and H3 K9Me3. However, models for changes in histone marks during cell cycle progression seem to differ depending on the model system employed [40,41].

Our model system, rat hepatocytes in primary culture, represents one in which histone modifications are established in these cells’ mature nucleosomes. The cell cycle changes that we found to be significant in cultured adult hepatocytes were restricted to H3.1/3.2 and H4. More specifically, there was a decline in H4 K20Me3 after 48 and 72 hr exposure to EGF plus insulin. This effect, consistent with changes in G2/M, appeared to be accompanied by a corresponding increase in abundance of H4 K20Me1. Changes in the methylation of lysine 20 have been shown to occur during the cell cycle [42,43], with monomethylation peaking during mitosis in HeLa cells [44]. However, alteration of the kinetics of K20 methylation in HeLa cells did not affect cell cycle progression suggesting that this mark may not be critical to mitotic regulation [44].

In addition to the cellular heterogeneity of liver tissue, our study has several other limitations. First among these is the bottom-up approach to proteomic analysis. Our data do not allow for the detection of combinatorial differences at sites that do not co-occur on specific peptides. Complex changes at multiple sites of histone modification may well result in a much greater number of histone proteoforms than is apparent from our data. Secondly, we assessed relative abundance of specific marks across various conditions. These results should not be interpreted as indicating precise stoichiometry. Finally, our data address histone PTMs across the entire population of nucleosomes, thus providing no information regarding the significance of these modifications for chromatin accessibility or regulation of gene expression. Nonetheless, our findings indicate that analyses of histones derived from *in vivo* or *in vitro* sources may reflect changes that are rapidly induced in response to alterations in the cellular milieu. This, in turn, has implications for studies aimed at assigning biological significance to histone modifications in tumors versus cancer cells, the developmental behavior of stem cells, and the attribution of changes in histone PTMs to altered cell metabolism.

## Supporting information

S1 Table Histone peptides, sites and site modifications quantified through the multiple reaction monitoring (MRM) analysis. The three spreadsheets within this file provide the histone modifications monitored during the experiments reported in this manuscript. The designation “H3.1/3.2” in this and in S2–S5 Tables reflect the fact that this peptide is present in both histones, so our analysis cannot differentiate between the two.
(XLSX)

S2 Table Peak areas derived from skyline peak area assignments. The individual spreadsheets contained within this file include peak areas generated in all of the analyses reported in this manuscript.
(XLSX)

S3 Table Relative abundance of all detected histone modifications. The individual spreadsheets contained within this file include relative abundance data generated in all of the analyses reported in this manuscript.
(XLSX)

S4 Table Relative abundance data for liver, freshly isolated adult hepatocytes, and cultured adult hepatocytes. (XLSX)

S5 Table Significant histone PTMs identified by ANOVA in the comparison of liver, freshly isolated adult hepatocytes, and cultured adult hepatocytes. The second, third and fourth spreadsheets contain significant peptides with adjusted p-values that were identified in the pair-wise analyses indicated in the spreadsheet titles.
(XLSX)
